# Immunotherapeutic Approach for Improving the Efficacy of a Novel Subunit Vaccine Against SARS-CoV-2 by Cytotoxic T-Lymphocytes (CTL) Epitopes

**DOI:** 10.1155/sci5/6025826

**Published:** 2025-05-26

**Authors:** Momina Javaid, Mahnoor Sagheer, Muhammad Zafar Saleem, Nazim Hussain, Nayla Munawar

**Affiliations:** ^1^Center for Applied Molecular Biology (CAMB), University of the Punjab, Lahore 54357, Pakistan; ^2^Department of Chemistry, College of Science, United Arab Emirates University, Al-Ain 15551, UAE

**Keywords:** CTL epitopes, MHC-1 receptor, SARS-CoV-2, subunit vaccine, TLR-8 receptor

## Abstract

The present study aimed to employ a diverse range of immunoinformatics and in vitro techniques to construct and validate a potentially active multiepitope subunit vaccine against SARS-CoV-2 using cytotoxic T-lymphocyte epitopes. To design the vaccine, a library of antigenic, nonallergic, and immunogenic epitopes of the spike protein was prepared. To improve the immunogenicity and safety of the final subunit vaccine, a sequence comprising three antigenic and nontoxic CTL epitopes was selected. To predict the tertiary structure of the vaccine, docking studies manipulating human major histocompatibility complex 1 (MHC-1) and Toll-like receptor-4 and Toll-like receptor-8 (TLR-4 and TLR-8) receptors were carried out. The consistency of the vaccine's binding to the selected receptors was confirmed by molecular dynamics (MD) simulations. In addition, the cloned vaccine was introduced into a bacterial culture, and its expression and antigenicity were assessed using SDS-PAGE and Western blotting, respectively. The vaccine design revealed a strong affinity for the TLR-8 and MHC-1 receptors, as evidenced by molecular docking analysis. The MD simulations conducted in specific systems yielded further data supporting the robust and enduring binding of TLR-8 and MHC-1 receptors to CTL epitopes. The bacterial cells harboring the vaccine sequence demonstrated robust production of the vaccine protein upon induction with IPTG. In addition, Western blotting demonstrated the antigenic properties of the vaccine protein. Computational and in vitro analyses suggested that the designed multiepitope subunit vaccine is stable and can induce specific immune responses against SARS-CoV-2.

## 1. Introduction

Vaccination has been playing a pivotal role in safeguarding humankind against various deadly pathogens, including viruses, parasites, and bacteria. Indeed, the discovery and production of vaccines is one of the most impactful investments for mankind that has ever made for its own protection [1]. The production of reliable and effective vaccines that curtail mortality and morbidity rates is considered the most significant advancement of the 21st century [2]. Ever since the end of 2019, the world has experienced widespread infection caused by SARS-CoV-2 [3]. Moreover, many pharmaceutical and nonpharmaceutical companies have expanded their ample time and money to find a cure for illness caused by the virus [4].

The pathogen responsible for COVID-19 is a member of the beta coronavirus genus within the Coronaviridae family [5]. The structure of SARS-CoV-2 consists of a 180–200 kDa spike (S) protein that plays a role in viral transmission [6]. It is composed of an external N-terminus fixed in the viral membrane, a transmembrane (TM) domain, and a brief C-terminal region [7]. In its resting state, the S protein has a stable structural configuration. However, during viral attack, the S protein undergoes significant conformational changes that allow entry of the virus into the host cell [8]. Proteases in infected target cells split the S protein into S1 and S2 components [9–12]. The transmission of disease from one human to another occurs via direct contact, respiratory droplets, or fomites, leading to a broad spectrum of symptoms, including cough, fever, muscle pain, headache, vomiting, and diarrhea [13]. The SARS-CoV-2 strain is responsible for the COVID-19 pandemic, and the need for flexible platform technologies that allow for the rapid manufacture of vaccines specific for developing viral infections has increased [14].

Its antigenicity is credited to the structural proteins of the virus, particularly the S protein; unsurprisingly, numerous T- and B-cell epitopes have been found in this protein. The relevance of S protein to infection and pathogenesis makes this protein an appropriate target for the development of SARS-CoV-2 vaccines. During the course of infection, the major viral antigen is neutralized by antibodies [15]. Structural proteins contain epitopes that are identified by specific receptors on T-cell or B-cell surface. These receptors are among the antigenic determinants that can initiate cellular immune responses [4]. Consequently, identification and analysis of epitopes is necessary for the development of epitope-based vaccines and diagnostic techniques [13].

Epitope-driven vaccines, founded on immune-based principles, provide novel ways to combat infectious agents, including quickly modifying or mutating pathogens, by focusing on epitopes that are conserved and generating competent, highly specific immune responses. Very few articles have been published on COVID-19 subunit vaccines [16]. Renhe Yan et al. [17] utilized human embryonic kidney-293T cells to develop a quick and efficient production method for a new subunit vaccine candidate. The immunogenicity of the vaccine candidates was assessed in the pigs. This study revealed that a vaccination candidate centered on the Delta variant S protein could deliver effective and wide defense against the prototype SARS-CoV-2 and variants of concerns (VoCs). Additionally, the effective and durable creation of recombinant proteins utilizing HEK-293T cells might be useful as a universal platform for future vaccine development [17]. Prior research has shown that COVID-19 patients requiring intensive care have a decreased cytotoxic capacity and exhibit preferential depletion of CD8^+^ T cells or cytotoxic T-lymphocyte (CTL)–specific lymphopenia, suggesting the significance of these cells in the immune response [18].

The use of modern in silico immunoinformatic technologies, such as predicting and screening candidate epitopes and creating efficient vaccines in contrast to illnesses, minimizes the load of experimental immunity on the model organism [4]. These methods provide accurate, quick, and cost-effective methods for creating possible multiepitope vaccines contrary to infectious diseases [19]. However, developing a subunit vaccine will be more reliable than using complete proteins and attenuated viruses, which may cause sensitization and other immunological reactions, possibly including localized redness, discomfort, tenderness, or an increase in body temperature after immunization [19]. Furthermore, this technique offers various advantages, including the ability to tailor epitopes for increased potency, allow immune responses to conserved epitopes, and improve immunological profiles [18], all of which apply to diverse SARS-CoV-2 strains [20].

Bridging the earlier evidence requiring unique CD8^+^ T-cell responses in SARS-CoV-2 infections, the present study focused on the bioinformatics-based design and cloning of a multiepitope subunit vaccine targeting the SARS-CoV-2 S protein with an emphasis on CTLs followed by its expression in *E. coli* in vitro. The trRosetta server is a computational tool used to predict the high-accuracy protein structures, orientations, and inter-residue distances and model the 3-dimensional structures of protein providing insights into protein folding and function. Subsequently, molecular docking analyses were conducted to investigate the interaction or binding between the final vaccine design and key immunological receptors, including Toll-like receptor-4 (TLR-4), Toll-like receptor-8 (TLR-8), and major histocompatibility complex 1 (MHC-1). This study, taking into account protein structural variations, highlights the potential of vaccination capable of eliciting humoral and cellular immune responses to address a wide variety of variants.

## 2. Methodology

### 2.1. Retrieval and Antigenic Assessment of the S Protein Sequence

**Figure 1:**
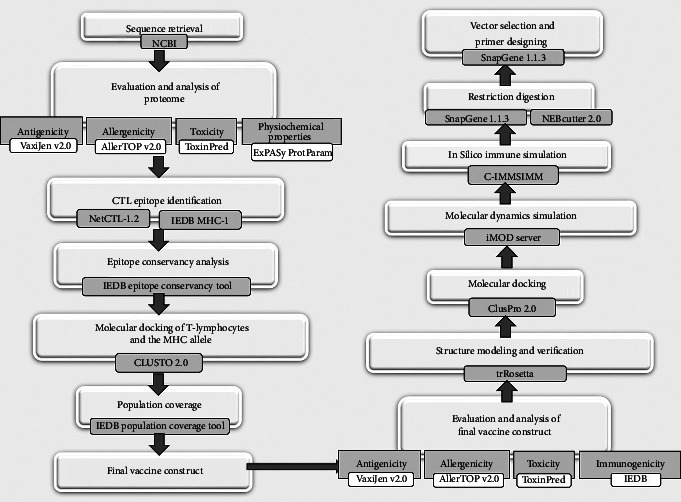
A bioinformatics flowchart exhibiting all the necessary steps used in designing subunit vaccine.

Using the accession number QZH77230.1, the sequence of the surface glycoprotein of SARS-CoV-2 was retrieved from the National Centre for Biotechnology Information (NCBI) (https://www.ncbi.nlm.nih.gov/). Moreover, 1273 amino acids (AAs) make up the protein sequence, which was subsequently saved in FAST-All (FASTA) format. This sequence was used for further protein analysis [21]. A bioinformatics flowchart showing all the necessary steps to design the subunit vaccine has been illustrated in Figure 1.

### 2.2. Evaluation and Analysis of the Antigenic Proteome

The VaxiJen v2.0 server (https://www.ddg-pharmfac.net/vaxijen/VaxiJen/VaxiJen.html) was used to assess the antigenicity of the SARS-CoV-2 surface glycoprotein [9]. VaxiJen utilized an unusual alignment-independent method that converts protein sequences into unchanging vectors of substantial AA properties using autocovariance (ACC) [22]. The protein sequence was pasted in the dialogue box provided in the web server, and the threshold was set to default. The allergenicity of the selected protein was assessed using AllerTOP v2.1 (https://www.ddg-pharmfac.net/allertop-test/) which uses an ACC transformation to turn proteins into homogeneous vectors and z-descriptors to represent the AAs in protein sequences [23]. ToxinPred (https://webs.iiitd.edu.in/raghava/toxinpred2/batch.html), a server used to design, predicts the toxic peptides by pasting the protein sequence in it [24]. The threshold value was set at a default value of 0.4. Similarly, the expert protein analysis system (ExPASy), a ProtParam tool (https://web.expasy.org/protparam/), which explores some features of the proteins, including molecular weight, theoretical pI, aliphatic index (AI), and AA composition [25].

### 2.3. Prediction and Assessment of CTL Epitopes

Vaccine design requires the accurate estimates of CTL epitopes. Above all, they can reduce the amount of experimental work required to find the epitopes. The web-based application called NetCTL could be used to predict the human CTL epitopes in any protein. It carried out this function through combining the MHC Class I affinity, TAP transport efficiency, and proteasomal cleavage predictions [26]. Both the NetCytotoxic T-lymphocyte epitope predictor (NetCTL-1.2) (https://services.healthtech.dtu.dk/services/NetCTL-1.2/) and the immune epitope database and analysis resource (IEDB) (https://tools.immuneepitope.org/mhci/) were also used to predict the CTL epitopes [27]. The online server was opened, and protein sequence was pasted in the dialogue box provided in both tools. According to the results of the NetCTL and IEDB MHC-1-binding prediction servers, the overlapping epitopes were carefully chosen for further analysis. The selected epitopes were subjected to antigenicity test using the VaxiJen v2.0 and toxicity evaluation using the ToxinPred server with the default parameters [28]. Afterward, the remaining nontoxic epitopes were subjected to an allergenicity test using AllerTop v2.0 server. The Class 1 immunogenicity tool of the IEDB analysis resource was used to assess the immunogenicity of the selected epitopes [29]. Epitopes that had positive values for immunogenicity were considered for further study.

### 2.4. Epitope Conservancy Analysis

The conserved epitopes in an epitope-based vaccination offer more protection against a variety of strains [30]. Therefore, to assess the conservancy of the identified epitopes, the sequence of the SARS-CoV-2 surface glycoprotein was retrieved from the NCBI. These sequences were subsequently aligned using the IEDB epitope conservancy tool (https://tools.iedb.org/conservancy/) [30]. To predict the conservancy of the five selected epitopes, the conservancy analysis tool from the IEDB was utilized. The epitopes that had 100% maximum and minimum identity were chosen for final vaccine construction.

### 2.5. Molecular Docking of T-Lymphocytes and the MHC Allele

The computer approach that predicts the ligands' binding affinity to receptor proteins was performed to assess the affinity of CTL epitopes for their respective alleles, molecular docking [31]. The 3D structure of the MHC Class 1 allele (Protein Data Bank [PDB] ID: 2XPG) was obtained from the Research Collaboratory for Structural Bioinformatics Protein Data Bank (RSCB PDB) [32]. PEP-FOLD3 (https://bioserv.rpbs.univ-paris-diderot.fr/services/PEP-FOLD3/) is another online program that predicts the peptide structures based on AA sequences using a crude representation. It creates conformations about four successive residues using a probabilistic framework based on structural alphabet letters. The primary output is 3D models of the expected structures that could be interactively observed. The 3D structure of the selected epitopes was obtained by using the PEP-FOLD 3.5 [33]. The clustering program (ClusPro 2.0) (https://cluspro.org/login.php) server was utilized for docking analysis. The allele and 3 epitopes were docked separately by ClusPro 2. The ClusPro server (https://cluspro.org) is another tool utilized for protein–protein docking. With just two files in protein data bank format needed for basic operation, the service offers a straightforward home page. The elimination of unstructured protein regions, the application of attraction or repulsion, the consideration of pairwise distance restraints, the construction of homo-multimers, the consideration of small-angle x-ray scattering (SAXS) data, and the identification of heparin-binding sites are some of the more sophisticated options that ClusPro provides to alter the search. Depending on the situation, six distinct energy functions can be utilized [34]. The epitope–allele complex with the lowest docking energy was considered the best candidate for vaccine development. Later, Python Molecular (PyMOL) software, a graphical environment for modeling and visualization of molecules, was used to visualize the interactions involving the epitopes and the MHC-1 allele [35].

### 2.6. Population Coverage Analysis

Analysis of population coverage was used to evaluate the frequency of human leukocyte antigen (HLA) antibodies associated with the epitope of interest, thus revealing the global efficacy of the designed vaccine. The IEDB population coverage tool was used to assess the coverage of selected epitopes with their subsequent MHC-1 alleles [36]. The IEDB analysis resource population coverage tool was opened online. The area or population was selected which was “World” in our study. MHC Class 1 was selected as a calculation option. The selected epitopes were submitted along with their respective alleles in an online tool to calculate the population coverage to understand the global efficacy of the designed vaccine.

### 2.7. The Subunit Vaccine Construct Was Formulated as Follows

To create a subunit vaccination, epitopes that are antigenic in nature, not allergenic, not toxic, and immunogenic were evaluated and chosen. Among all selected epitopes, only three epitopes were finalized; these epitopes were in close proximity to protein sequence and fully conserved, thus generating a 122 AA long target sequence in the SARS-CoV-2 surface glycoprotein [29].

### 2.8. Anticipating the Antigenic, Toxic, Physicochemical, and Allergenic Characteristics of the Finalized Vaccine

To evaluate the stability of the designed subunit vaccine and its physiochemical properties, the antigenic nature, allergenicity, toxicity, and immunogenicity of the designed vaccine were predicted using the VaxiJen v2.0, AllerTop 2.0, ToxinPred, and IEDB immunogenicity analysis tools, respectively. The sequence of the designed subunit vaccine was put in the dialogue box for the predictions.

Additionally, the solubility of the designed subunit vaccine was analyzed using the Protein-Sol server (https://protein-sol.manchester.ac.uk/) [37]. The scaled solubility value (QuerySol) provided an indication of the predicted solubility. A scaled solubility value above 0.45 suggested that the protein was likely more soluble than the average soluble *E. coli* protein in the experimental solubility dataset [38], while a value below 0.45 indicated lower solubility.

### 2.9. Structure Modeling and Verification

PSI-blast-based secondary structure PREDiction (PSI-PRED) (https://bioinf.cs.ucl.ac.uk/psipred/) addressed the strand, coil, and helix creation in the vaccine structure. The PSI-PRED protein analysis workbench is a web service providing a diverse suite of protein prediction, and annotation tools focused on structural annotations of proteins [39]. The query sequence was put in the online server for predictions. The trRosetta server (https://yanglab.qd.sdu.edu.cn/trRosetta/) was utilized to produce the various tertiary structure models of the vaccine. Structural models were downloaded into the PDB file. To find the best model, validation was performed via the single assay-wide variance experimental server (SAVES) (https://saves.mbi.ucla.edu/), which includes various tools, such as error function for residue-level assessment of protein structures (ERRAT) [40], to assess nonbonded atomic interactions; Verify-3D [41], for analyzing the compatibility of the 3D protein model with AA sequences; and PROCHECK, for analyzing the stereochemical and geometrical constraints of the 3D protein model by Ramachandran plot analysis [42]. The Galaxy Refine server (https://galaxy.seoklab.org/cgi-bin/submit.cgitype=REFINE) first rebuilds all side-chain conformations and then repeatedly relaxes the structure using brief molecular dynamics simulations after side-chain repacking perturbations. The best structure was then selected after validation using the SAVES, as mentioned earlier. This technique can increase the global and local structure quality [43]. ProSA-web (https://prosa.services.came.sbg.ac.at/prosa.php), an interactive web service for the recognition of errors in three-dimensional structures of proteins, was also employed to estimate the Z-score of the protein structure [44]. The z-score assesses overall model quality by measuring the structure's total energy departure from an energy distribution derived from random conformations. Z-scores that are outside the range expected for native proteins indicate incorrect structures [44].

### 2.10. Molecular Docking Analysis of the Vaccine Peptide

The ClusPro 2.0 server was used to evaluate interactions among the designed vaccine model and between TLR-4, TLR-8, and MHC1 receptors. By using the PIPER docking algorithm, ClusPro v2.0 was used to predict protein–protein interactions [34]. The crystal structures of TLR-4 (PDB: 4G8A), TLR-8 (PDB: 3W3G), and MHC Class 1 (PDB: 2XPG) were retrieved from the RSCB PDB and exploited as receptors, while the vaccine molecule was exploited as a ligand in the analysis. PROtein binding enerGY prediction (PRODIGY) (https://rascar.science.uu.nl/prodigy/) is a set of online services that focus on predicting the binding affinity in the biological complexes and identifying the biological interfaces using the crystallographic data. PRODIGY protein–protein tool was selected while the PDB file of the docked complex was used as input while setting the temperature at 37°C. The resulting docked complexes were visualized with PyMOL.

### 2.11. Molecular Dynamic Simulation of the Subunit Vaccine

The iMOD (multipurpose normal mode analysis [NMA] in internal coordinates) (https://imods.iqf.csic.es/) server was used to carry out a molecular dynamic simulation of the receptor–vaccine complex [45]. PDB format of atomic coordinates was submitted to explore the collective motions of proteins and nucleic acids using NMA in internal coordinates (torsional space). iMOD server was used to analyze the deformability, NMA, eigenvalues, covariance matrix, mobility (NMA B-factors), and linkage matrix based on the 3D structure of the proteins. For dynamic simulation analysis, the vaccine–MHC1 complex, vaccine–TLR-4 complex, and vaccine–TLR-8 complex, which had the least binding energy, were used.

### 2.12. In Silico Immune Simulation

To analyze the real-life immunological and immunogenic responses of the developed vaccine, C-language version of IMMune system SIMulator (C-IMMSIMM) (https://kraken.iac.rm.cnr.it/C-IMMSIM/index.php) was utilized to execute an in silico immune simulation [46]. The server employs a position-specific scoring matrix (PSSM) that is drawn from machine learning techniques or the prediction of immune interactions. The lowest suggested interval between the first and second doses of the vaccine is 4 weeks [47], according to the currently used vaccines. Three injections containing one thousand antigen proteins and no LPS were given. The parameters for the random seeds, simulation volume, and simulation step were set to 12,345, 10 μL, and 100, respectively.

### 2.13. Restriction Digestion

SnapGene 1.1.3 and NEBcutter 2.0 (https://nc2.neb.com/NEBcutter2/noredir) were utilized for restriction mapping [37]. SnapGene was utilized to discover probable restriction enzyme cleavage sites in the vaccination sequence, supporting the identification of a suitable restriction site and expression vector. NEBcutter was utilized to identify all potential restriction sites in the vaccination sequence. The sequence in FASTA format was loaded into NEBcutter utilizing default settings.

### 2.14. Vector Selection and Primer Design

After the selection of appropriate restriction sites, an appropriate expression vector was selected using the SnapGene 1.1.3 tool. Forward and reverse primers were also designed with *Nde*I and *Bam*HI restriction sites at the 5′ ends, respectively, to facilitate the amplification of the target gene.

• Forward primer: CCATGGAGGTTTGATAACCCTGTCC
• Reverse primer: GAATTCTTCTGAGAGAGGGTCAAGT

### 2.15. In Silico Amplification and Cloning of the Final Vaccine

The SnapGene 1.1.3 tool was used for in silico amplification of the final vaccine sequence. In silico polymerase chain reaction (PCR) results were predicted by hybridizing the primer sets with the targeted sequence at a specific annealing temperature. This approach enables the rapid evaluation of designed primers against the target vaccine sequence, ensuring the identification of restriction sites in the PCR product.

After successful amplification of the designed vaccine sequence, the resulting sequence was cloned and inserted into a suitable bacterial expression vector, i.e., pET-28b (+), by utilizing the SnapGene 1.1.3 tool. The epitope-based subunit vaccine was subsequently cloned and inserted into the pET-28b (+) expression vector of *E. coli*. In silico cloning was performed by inserting the final vaccine sequence with the essential cleavage sites into the expression vector pET-28b (+) to assess its potential expression.

### 2.16. Gene Synthesis

The Centre for Applied Molecular Biology (CAMB) provided the gene construct, which included the target vaccine sequence with suitable and selected restriction sites in the suitable vector pET-28b (+). The resulting gene construct was named MJ-SPIKE.

### 2.17. Transformation of the MJ-SPIKE Plasmid Into the Top10 Strain

Both Luria–Bertani (LB) broth and LB agar (Cat. No. 244510, Thermo Fisher Scientific, USA) were prepared and sterilized by autoclaving before proceeding with the transformation. The LB broth was stored at 4°C for further use, and LB agar plates were prepared. For the selection of Top10 cells, the drug tetracycline (Cat. No. T7660, Sigma-Aldrich) (12.5 μg/mL) was added, while for vector selection, kanamycin (Cat. No. K1377, Sigma-Aldrich) (50 μg/mL) was added. Competent cells were prepared using glycerol (Reagent grade, Cat. No. G5516) stock of Top10 cells that were streaked on LB agar plates supplemented with the drug tetracycline and incubated in an oven for 16–18 h at 37°C. The competent cells among the Top10 cells were prepared by the calcium chloride method [48], which included the inoculation of a single colony into 5–10 mL of LB broth containing tetracycline. The culture was incubated at 37°C for 3–4 h in a shaking incubator until it reached the optical density (OD_600_) of about 0.4–0.6. The cells were transferred to a sterile centrifuge tube and chilled on ice for about 15 min. The cells were then subjected to centrifugation at 8000 rpm for 10 min at 4°C. The supernatant was discarded. The pellet was resuspended in 1 mL sterile and cold 0.1 M CaCl_2_ (Cat. No. 209139, Sigma-Aldrich). The mixture was incubated on ice for 30 min, by gently inverting the tube every 5–10 min. The cells were centrifuged at 8000 rpm for 10 min at 4°C, while the pellet was resuspended in a smaller volume (100–200 μl) of cold 0.1 M CaCl_2_. The freshly prepared competent cells were then used for the transformation of the target plasmid MJ-SPIKE using the heat-shock method [48].

Moreover, 3 μl of target plasmid MJ-SPIKE was added to competent cells followed by incubation on ice for 30 min. The mixture was incubated at 42°C for 1 min and then immediately placed back on ice for a heat shock. Finally, 1 mL of LB broth was added to it and incubated at 37°C with agitation for 16–18 h which was used for obtaining isolated colonies on agar plates [48].

### 2.18. Screening and Confirmation of the Transformed Colonies

The next day, colonies that formed on the plates showed successful transformation of our target plasmid into the Top10 cells of *E. coli*. Colonies were selected for the confirmation of the transformed plasmid. Colonies were picked using sterile toothpicks, inoculated in LB broth supplemented with tetracycline and kanamycin and incubated at 300 rpm overnight at 37°C. A Plasmid Miniprep Kit (Cat. No. K0504, Thermo Fisher Scientific) was used for plasmid isolation. The plasmid isolation protocol was followed as described in the manual provided with the kit. The extracted MJ-SPIKE plasmid was amplified using specific primers (Forward and Reverse) in a thermocycler (ABI; Gene Amp 9700 system) under the following conditions: initial denaturation at 95°C for 5 min, followed by 35 cycles of denaturation at 95°C for 30 s, annealing at 52°C for 30 s, and extension at 72°C for 1 min, followed by final extension at 72°C for 10 min. The reaction mixture was prepared in individual 0.2-mL tubes for PCR amplification (Table S1). The amplification of the MJ-SPIKE gene was performed via PCR (Table S2). After the run, the gel was examined on a 1% agarose (Sigma-Aldrich) gel with TAE buffer (1x).

### 2.19. Transformation of the MJ-SPIKE Plasmid Into BL21(D3) Cells

After successful transformation and confirmation of positive *E. coli* Top10 colonies, the MJ-SPIKE plasmid was transformed into expression host cells, i.e., *E. coli* BL21(DE3) cells. Competent BL21(D3) cells were prepared using glycerol (Reagent grade, Cat. No. G5516) stock of BL21(D3) cells that were streaked on LB agar plates supplemented with no drug and incubated in an oven for 16–18 h at 37°C. The competent cells among the BL21(D3) cells were prepared by the calcium chloride method which included the inoculation of a single colony into 5–10 mL of LB broth having tetracycline [48]. The culture was incubated at 37°C for 3-4 h in a shaking incubator until it reached the OD_600_ of about 0.4–0.6. The cells were transferred to a sterile centrifuge tube and chilled on ice for about 15 min. The cells were then subjected to centrifugation at 8000 rpm for 10 min at 4°C, and supernatant was discarded. The pellet was resuspended in 1-mL sterile and cold 0.1 M CaCl_2_ (Cat. No. 209139, Sigma-Aldrich). The mixture was incubated on ice for 30 min, by gently inverting the tube every 5–10 min. The cells were centrifuged at 8000 rpm for 10 min at 4°C, while the pellet was resuspended in a smaller volume (100–200 μl) of cold 0.1 M CaCl_2_. The freshly prepared competent cells were then used for the transformation of the target plasmid MJ-SPIKE using the heat-shock method [48].

Moreover, 3 μl of target plasmid MJ-SPIKE was added in competent cells followed by incubation on ice for 30 min. The mixture was then incubated at 42°C for 1 min and then immediately placed back on ice for heat shock. 1 mL of LB broth was added to it and incubated at 37°C with agitation for 16–18 h which was then used for obtaining isolated colonies on agar plates [48].

### 2.20. Bacterial Expression (*E. Coli* BL21(D3)) of MJ-SPIKE Vaccine

For the protein expression analysis, a positive colony was picked and resuspended in 10 mL of LB broth containing the antibiotic kanamycin (50 μg/mL), followed by incubation at 37°C until the OD_600_ reached 0.4–0.8. The culture was diluted to a ratio of 10:100 and incubated at 37°C in a shaking incubator for three to 4 hours. Induction was subsequently carried out for 18 h at 37°C using 100 μL of 1 M IPTG (Thermo Fisher Scientific) stock solution (with a final working concentration of 1 mM). Protein expression was determined using a Coomassie blue–stained protein gel (sodium dodecyl sulfate-polyacrylamide gel electrophoresis [SDS-PAGE]) and a Western blot. For this purpose, an uninduced sample of approximately 500 μL was saved before induction, while induced culture samples (500 μL) were also removed. The soluble and insoluble fractions were separated via SDS-PAGE for protein analysis [49].

### 2.21. SDS-PAGE

To prepare the samples for SDS-PAGE, 1.5 mL of uninduced and induced cultures was centrifuged at 14,000 rpm for two minutes. The resulting pellets were then resuspended in 100 μL of 1x PBS buffer and 35 μL of 6x SDS loading dye. One hundred microliters of whole extract and a tiny amount of insoluble fraction were combined with 100 μL of 1x PBS buffer and 35 μL of 6x SDS loading dye. After removing 65 μL of the supernatant, the soluble fraction was directly mixed with 35 μL of 6x SDS loading dye. The samples were then placed on a hot plate, incubated for 10 min at 95°C, and quickly transferred to ice. After 1 minute on ice, the samples were centrifuged at 14,000 rpm for 1 minute and then were ready to load [49]. A resolving gel (12.5%) and a stacking gel (4%) were made to prepare an SDS-PAGE gel, as shown in Tables S3 and S4. After the apparatus was set, all the reagents were added in sequence. After the run, the gel was carefully removed from the plates and stained using a staining solution containing Coomassie brilliant blue as the primary dye. The gel was preserved the next day with a destaining solution (acetic acid and methanol). The expressed proteins were then visualized [50].

### 2.22. Western Blotting

For Western blotting, specific antibodies are used to recognize proteins that have been resolved based on size by gel electrophoresis [51]. We expressed the MJ-SPIKE vaccine protein again, preparing the samples in the same way as for the first gel. The proteins were separated via 12.5% SDS-PAGE with an 80-V constant current in a mini-gel device from Bio-Rad for two and a half hours while a 250-kDa prestained protein marker (Cat. No. 26616, Thermo Fisher Scientific) was used as a standard marker. Following electrophoresis, the proteins were transferred to a PVDF membrane (Cat. No. IPVH00010, Merck Millipore, Germany). The transfer was carried out under standard conditions (100 V for 1 h) to ensure efficient transfer of proteins from the gel to the membrane. The blot was washed with 1x PBST buffer twice for 15 min to remove the residual SDS and unbound proteins. To rule out the nonspecific binding of antibodies, the membrane was blocked by incubating it with skimmed milk in 1x PBST overnight. The membrane was subsequently treated with the anti-His-tag primary antibody (Cat. No. 11175-1-AP, ProteinTech, Chicago, USA) to detect our tagged protein. Later, the membrane was washed with 1x PBST for 15 min to remove any unbound primary antibodies. Next, the membrane was incubated with AP-conjugated goat–anti-rabbit IgG secondary antibodies (Cat. No. 1706518, Bio-Rad Laboratories, USA) which were diluted at 1x PBST at 1:15,000. The membrane was incubated for an hour at 30°C. The membrane was washed twice at room temperature with 1x PBST wash buffer for 15 min with gentle agitation after incubating it. For the color reaction, a 10-mg NBT/BCIP tablet (Sigma-Aldrich) was dissolved in 10 mL of water and incubated with the membrane for 1 h. The color reaction stopped after 60 min of incubation at 37°C. The blots were subsequently dried and analyzed [52].

## 3. Results

### 3.1. Retrieval and Antigenic Assessment of the S Protein Sequence

The proteome of the surface glycoprotein of the SARS-CoV-2 virus included 1273 AAs and was retrieved from the NCBI in FASTA format as described (see Table S5); this information included the corresponding accession number, length of AAs, and antigenic properties.

### 3.2. Evaluation of the Antigenic Proteome

The proteome of the SARS-CoV-2 surface glycoprotein was analyzed with VaxiJen v2.0, AllerTOP2.0, and ToxinPred 2.0 for the prediction of antigenicity, allergenicity, and toxicity, which predicted the proteome to be antigenic, nonallergen, and nontoxic, respectively, as mentioned in the table. The physiochemical properties of the selected proteomes are listed in Table S6.

### 3.3. Prediction and Assessment of CTL Epitopes

Five overlapping epitopes were identified and shortlisted for their binding affinity to MHC-I supertype alleles based on their IC_50_ value and low rank. VaxiJen v 2.0 and ToxinPred servers indicated that all five selected epitopes were antigenic (keeping the threshold value 0.4 and model organism as virus) and nontoxic. Furthermore, these epitopes were evaluated by AllerTOP v2.0 as nonallergenic, while IEDB Class 1 immunogenicity tool identified them as immunogenic (Table 1).

### 3.4. Epitope Conservancy Analysis

Of the five epitopes, three epitopes showed 100% identity and were thus selected for the final subunit vaccine (Table 2).

### 3.5. Molecular Docking of T-Lymphocytes and the MHC Allele

**Figure 2:**
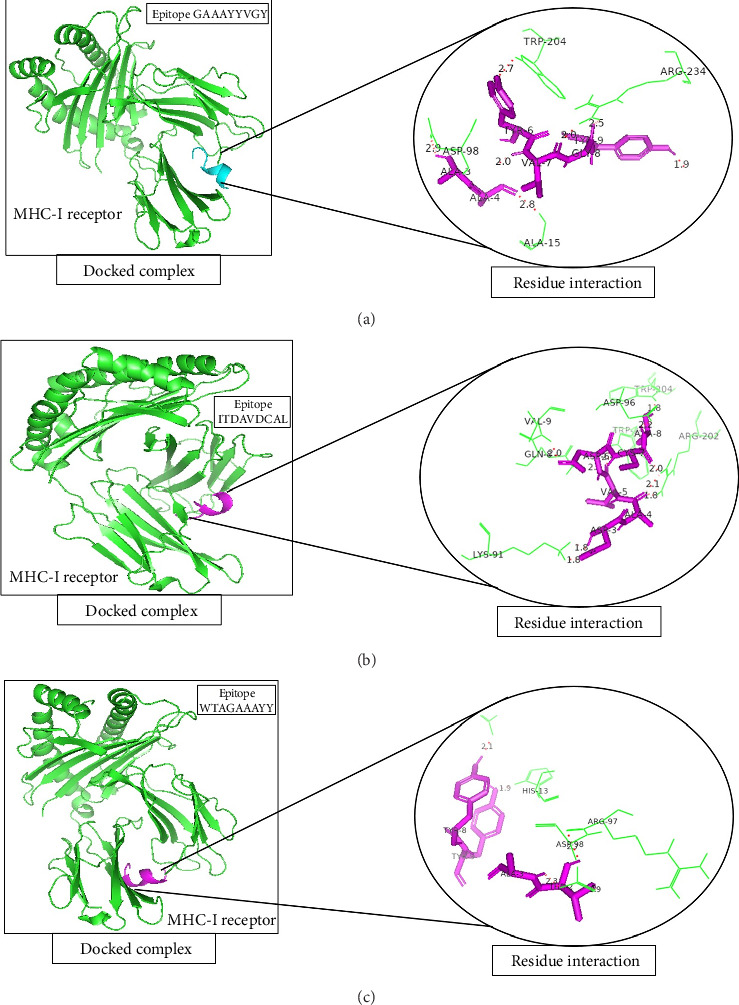
Molecular docking of selected CTL epitopes with the MHC-1 allele visualized via PyMOL. (a): Docked complex of MHC-1 (green) with the epitope GAAAYYVGY (magenta) along with a zoomed-in view of residue interactions within the docked complex. (b) Docked complex of MHC-1 (green) with the ITDAVDCAL epitope (magenta) along with a zoomed-in view of residue interactions within the docked complex. (c) Docked complex of MHC-1 (green) with the epitope WTAGAAAYY (magenta) along with a zoomed-in view of residue interactions within the docked complex.

A total of 5 bonds were found in the dock complex of the CTL epitope GAAAYYVGY and MHC-1; 8 bonds were found in the CTL epitope ITDAVDCAL and MHC-1; and 3 bonds were found in the CTL epitopes WTAGAAAYY and MHC-1, as shown in Figure 2.

### 3.6. Population Coverage Analysis

To forecast population coverage using the specified T-lymphocyte epitopes, IEDB population coverage analysis was used. Our vaccination protocol accounted for 25.61% of the global population's T cells (Figure S1).

### 3.7. The Subunit Vaccine Construct Was Formulated as Follows

After 3 CTL epitopes were selected, a proteomic region that included all three selected epitopes and consisted of 122 AAs was chosen for the final subunit MJ-SPIKE vaccine design.

### 3.8. Characteristics of the Finalized MJ-SPIKE Vaccine

Several features were evaluated to determine whether the MJ-SPIKE vaccine was suitable for human use. The VaxiJen, AllerTOP, and ToxinPred servers predict that the vaccine is antigenic, nonallergenic, and nontoxic in nature. ExPASy ProtParam showed that the subunit vaccine has a molecular weight of 13,648.51 Da, and its isoelectric point (5.57 pI) suggests that it is mildly acidic. The instability index was predicted to be 26.11, indicating that the vaccination will be stable. The AI of 91.97 denotes thermostability. The computed grand average of hydropathy (GRAVY) value of −0.172 indicated that the vaccine peptide was hydrophilic (Table S7). The Protein-Sol server estimated the scaled solubility to be 0.369, indicating that the solubility of the vaccine was slightly low (Figure S2).

### 3.9. Structure Modeling and Verification

**Figure 3:**
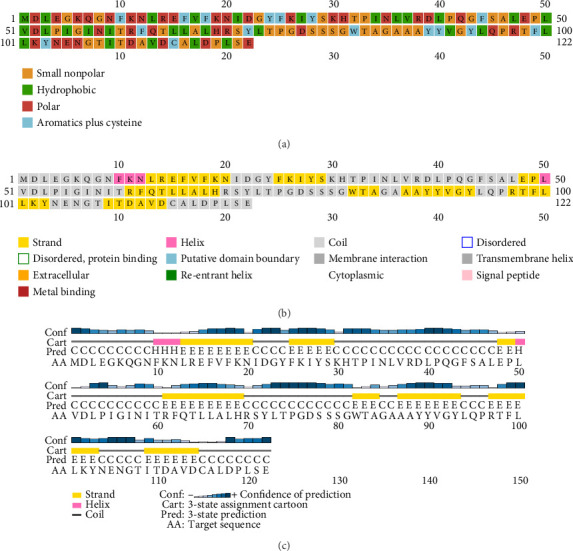
Secondary structure prediction of vaccines using PSI-PRED. (a) PSI-PRED showing polar, nonpolar, and hydrophobic interactions. (b) PSI-PRED showing strand, coil, and helix formation within the vaccine. (c) PSI-PRED image showing the 2D structure of the vaccine.

PSI-PRED, which was utilized to analyze the secondary structure of the vaccine, measures the number of AAs in the alpha helix, the development of coils, and the beta sheet inside the vaccine structure [41]. The vaccine was made up of polar, nonpolar, and hydrophobic compounds.  Figure 3 shows the protein analysis of the different kinds of strands, coils, and helices.

**Figure 4:**
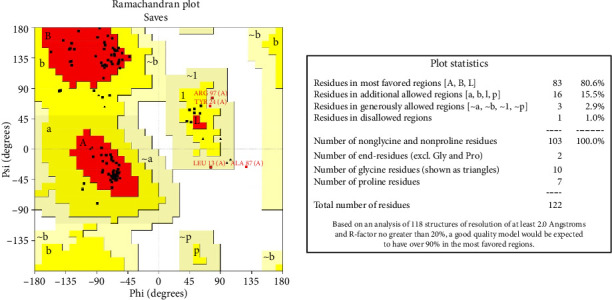
PROCHECK statistics of the selected model showing Ramachandran plot statistics.

Five distinct 3D models of the final subunit vaccine were produced using the trRosetta web server. Based on the highest calculated TM score and the authentication scores from the Verify-3D, ERRAT, PROCHECK, and ProSA-web servers, the best model among them was chosen (Figure 4 and Figure S3).

### 3.10. Molecular Docking of the Vaccine Peptide

**Figure 5:**
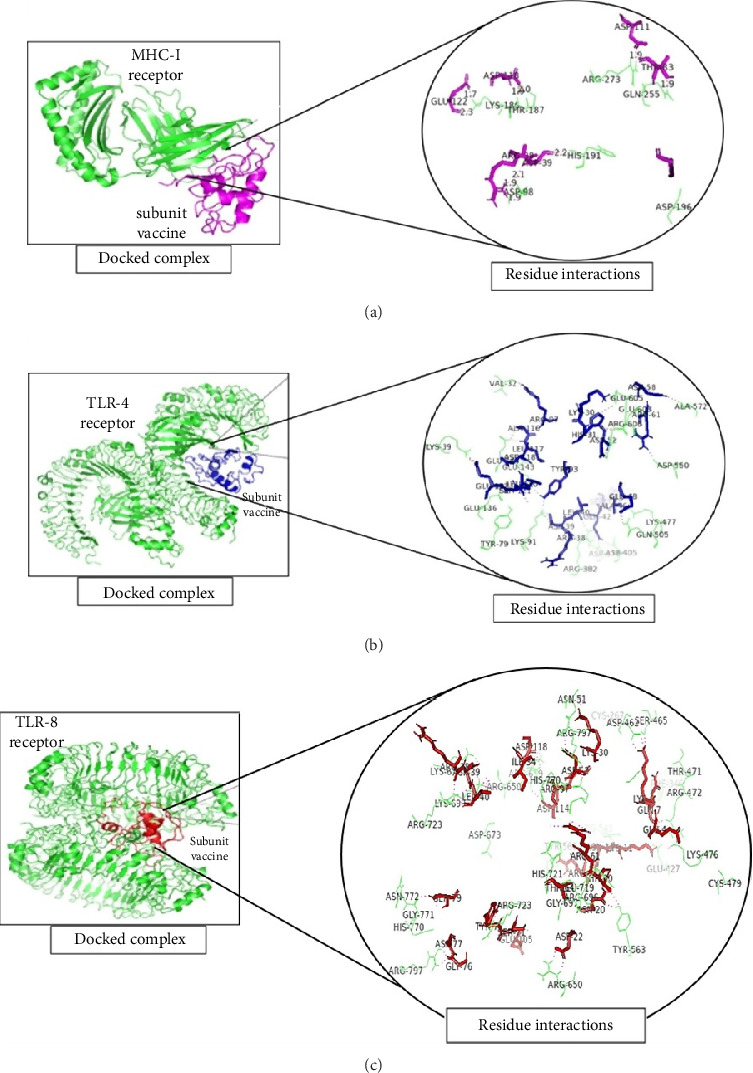
Molecular docking of the subunit vaccine with the receptors MHC-1, TLR-4, and TLR-8 visualized on PyMOL. (a) Docking complex of the subunit vaccine and the MHC-1 receptor along with a zoomed-in view of residue interactions within the complex. (b) Docked complex of the subunit vaccine and TLR-4 receptor along with a zoomed-in view of residue interactions within the complex. (c) Docked complex of the subunit vaccine and TLR-8 receptor along with a zoomed-in view of residue interactions within the complex.

To investigate the interactions between the vaccine model and TLR-4, the TLR-8 and MHC-1 receptor ClusPro servers were utilized for molecular docking. After the prediction of the MJ-SPIKE vaccine model using the trRosetta server and structural refinement, the structures of TLR-4, TLR-8, and MHC-1 were retrieved from the PDB with the IDs 4G8A, 3W3G, and 2XPG, respectively. The lower the binding energy is, the greater the stability of the docked complex. Thus, the selection of the minimum binding energy model was a crucial step. The subunit MJ-SPIKE vaccine exhibited the strongest interaction with the TLR-8 receptor, with a minimum energy value of −1062.4. Likewise, after docking with MHC-1, the model selected for analysis had the smallest energy value of −836.9. The lowest energy score for the interaction between TLR-4 and the subunit MJ-SPIKE vaccine was −759.8. The interaction between MHC-1 and the subunit MJ-SPIKE vaccine indicates a total of 9 hydrogen bonds between the active residues of MHC-1 and the subunit MJ-SPIKE vaccine construct, including Lys-186, Thr-187, Asp-98, His-191, Gln-255, Arg-279, and Asp-196 in the receptor and Glu-122, Asp-418, Arg-38, Asp-39, Asp-111, and Thr-83 in the vaccine sequence, as shown in Figure 5(a). The interaction between TLR-4 and the subunit MJ-SPIKE vaccine indicated a total of approximately 21 hydrogen bonds between the active residues of TLR-4 and the subunit MJ-SPIKE vaccine construct, including Tyr-79, Lys-91, Glu-136, Lys-39, Val-32, Arg-382, Asp-405, Gln-505, Lys-477, Asp-550, Ala-572, Glu-605, Glu-603, Arg-608-Glu-143, and Glu-144 in the receptor and Arg-38, Asp-39, Leu-40, Gln-42, Ala-46, Glu-48, Tyr-93, Asp-118, Leu-117, Arg-96, Ala-116, His-31, Lys-30, Asp-52, and Asn-58 in the vaccine sequence, as shown in Figure 5(b). The hydrogen bonds formed by TLR-8 include Ala-485, Glu-427, Tyr-563, Lys-476, Asp-462, Arg-810, Asp-72, Asn-51, Arg-797, Arg-797, Arg-650-Lue-748, and Ser-744. In contrast, the residues involved in hydrogen bonding with TLR-8 include Lys-6, Glu-4, Arg-696, Gly-76, Arg-61, Thr-60, and Arg-14, as shown in Figure 5(c). The hydrogen bonds strongly polarize the vaccine design with all three receptors, resulting in the downstream signaling cascade necessary to stimulate immune responses.

The PRODIGY online server was subsequently used to analyze the positions at which the dissociation constants and binding affinities were docked at approximately 37°C with the highest cluster member, which yielded G values of −8.0, −14.0, and −11.9 for the MJ-SPIKE vaccine–TLR-4 receptor, MJ-SPIKE vaccine–TLR-8, and MJ-SPIKE vaccine–MHC1, respectively, as shown in Table S8. All the interactions were determined to be energetically viable. This finding indicates the specificity of the docked model. The low dissociation constant and negative Gibbs free energy make this clear.

### 3.11. Molecular Dynamics Simulation

**Figure 6:**
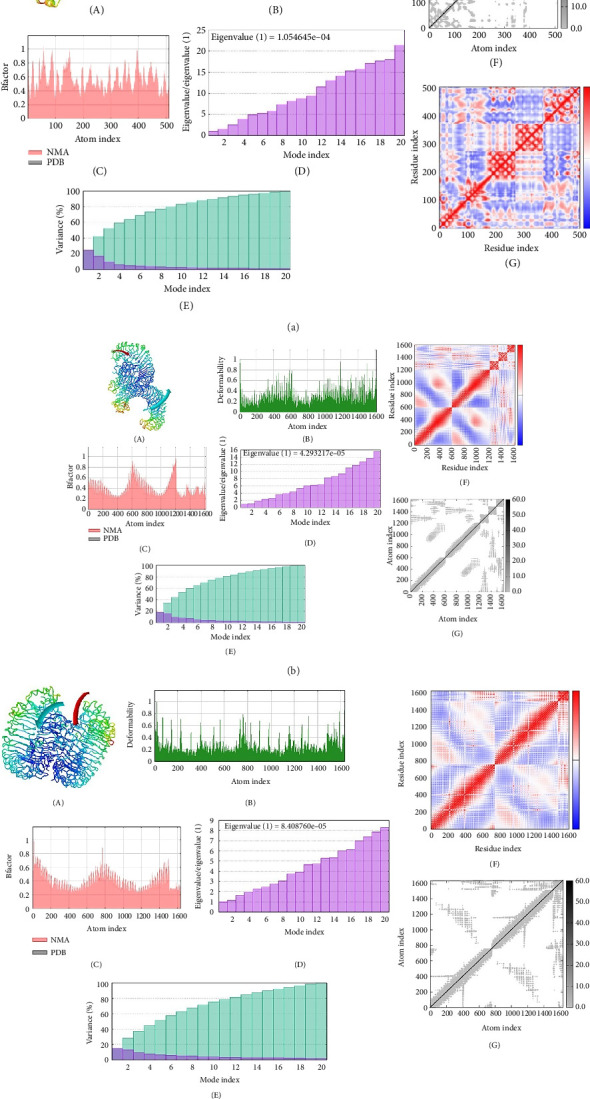
(a) Molecular dynamics simulation of the MHC-1 and subunit vaccine docked complex. (A) Vaccine–MHC-1 complex. (B) A simulation of deformability that shows the hinges—areas that are highly deformable. (C) B-factor values, which are determined via normal mode analysis and indicate the degree of uncertainty for every atom. (D) The docked complex's epigenetic value, which shows the force required to distort the structure. (E) The variance corresponding inversely to the epigenetic value of each normal mode. (F) The elastic network model, in which stiffer springs are shown by deeper grays, postulates a connection between atoms and springs. (G) Covariance matrix between pairs of residues. (b) Molecular dynamics simulation of TLR-4 and the subunit vaccine docked complex. (A) Vaccine–TLR-4 complex. (B) A deformability simulation displaying the hinges or highly deformable regions. (C) B-factor values, which show the level of uncertainty for each atom and were ascertained using normal mode analysis. (D) The epigenetic value of the docked complex, indicating the force necessary to deform the structure. (E) Variance that is inversely related to each normal mode's epigenetic value. (F) Covariance matrix between pairs of residues, where the colors blue, white, and red represent anticorrelation, no correlation, and correlation, respectively. (G) The elastic network model postulates a relationship between atoms and springs, with stiffer springs represented by deeper grays. (c) Molecular dynamics simulation of TLR-8 and the subunit vaccine docked complex. (A) Vaccine–TLR-8 complex. (B) A deformability simulation displaying the hinges or highly deformable regions. (C) B-factor values, which show the level of uncertainty for each atom and were ascertained using normal mode analysis. (D) The epigenetic value of the docked complex, indicating the force necessary to deform the structure. (E) Variance that is inversely related to each normal mode's epigenetic value. (F) Covariance matrix between pairs of residues, where the colors blue, white, and red represent anticorrelation, no correlation, and correlation, respectively. (G) The elastic network model postulates a relationship between atoms and springs, with stiffer springs represented by deeper grays.

The IMOD server critically analyzes structure by modifying complex force fields involving numerous time intervals. The selected MJ-SPIKE vaccine model was stable, as depicted by molecular dynamics simulations, as shown in Figures 6(a), 6(b), 6(c).

### 3.12. Immune Simulation

**Figure 7:**
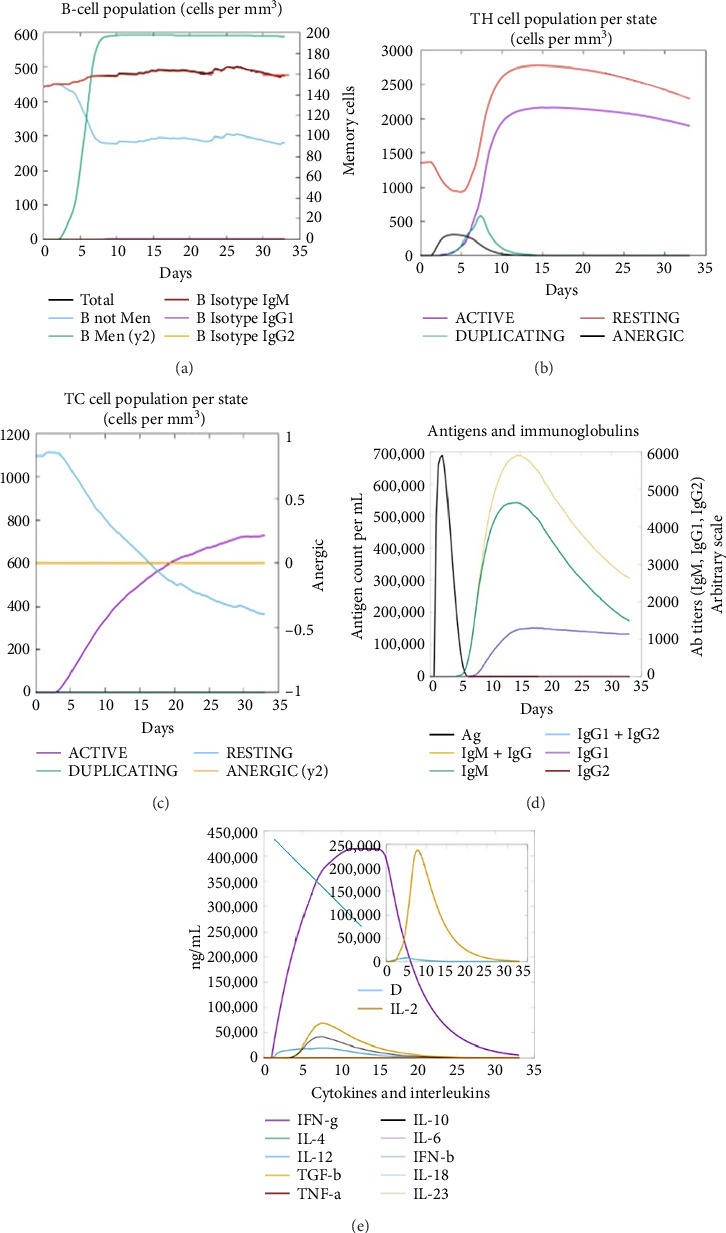
Immune simulation of the vaccine subunit (a) B-cell population, (b) T-cell population, (c) T-cell population per state, (d) antigens and immunoglobulins, and (e) cytokine and interleukin production.

The C-ImmSim server was utilized to investigate the simulated immunological response. IgM and IgG levels were elevated during the main reaction. Increases in B-cell populations and IgG1 + IgG2, IgM, and IgG + IgM antibody levels suggest the progression of immunological memory and competent immunity in response to further antigen exposure, as shown in Figures 7(a) and 7(d). The accompanying memory formation was accompanied by remarkable responses from the TH and TC cell populations, as shown in Figures 7(b) and 7(c). IgG1 levels increased after the third injection, while the TH cell population and IFN-γ concentration remained stable throughout the exposure period. IFN-γ and IL-2, which are thought to be the most important cytokines for an antiviral immune response, increased in tandem with the number of cytokines following each injection, as shown in Figure 7(e).

### 3.13. Restriction Digestion, In Silico Amplification, and Cloning

It is critical to understand each cleavage site before selecting the optimum expression vector. Figures S4 and S5 show the results of in silico amplification of the target sequence alongside each potential cleavage site in the sequence. These findings could provide some useful information on further downstream techniques, including vector selection.

**Figure 8:**
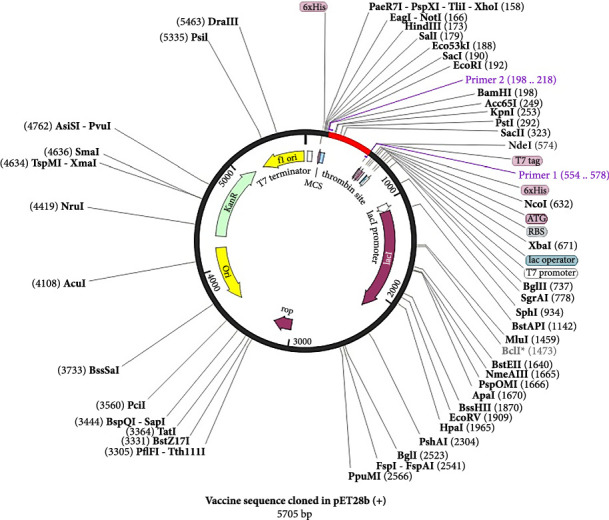
Cloning of the subunit vaccine sequence (red) in the pET-28 (+) expression vector (black).

**Figure 9:**
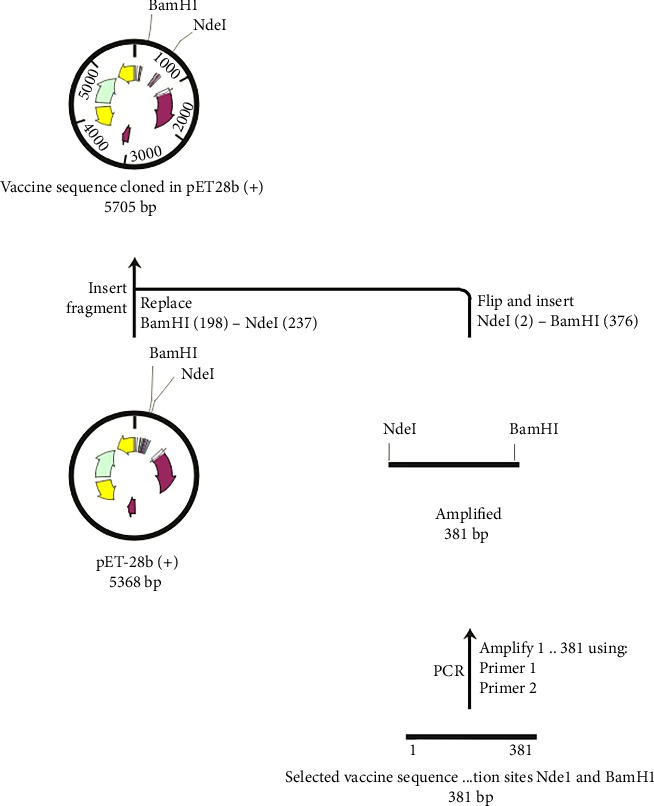
In Silico cloning map: The pET28b (+) vector and the chosen vaccine sequence were restriction digested using the *Nde*I 1 and *Bam*HI enzymes as part of the in silico cloning process.

**Figure 10:**
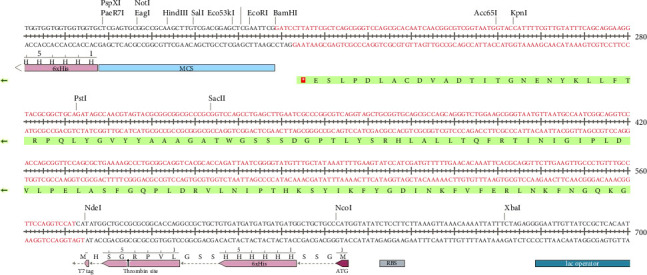
In-frame in silico expression of MJ-SPIKE vaccine: a zoomed-in view of the sequence around both the *Nde*I and *Bam*HI insertion sites, showing that the protein is in frame.

In the present investigation, the two developed primers efficiently bound to the template DNA, allowing for efficient amplification of the vaccine sequence. The whole MJ-SPIKE vaccine sequence was subsequently cloned and inserted into the pET-28b (+) vector using SnapGene software, as shown in Figure 8. The pET-28b (+) expression vector is represented in black, and the MJ-SPIKE vaccination sequence (381 bp) is highlighted in red. The efficacy of PCR is dependent on the primer used and the target sequence used for specific amplification. We can now forecast the theoretical success of PCR by developing exceedingly complex and accurate primers before starting expensive laboratory trials owing to advances in computer algorithms. Figures 8 and 9 show that inserting the selected sequence between the *Nde*l and *Bam*HI sites resulted in a 5705 bp clone, while Figure 10 shows the expressed protein in the frame. This in silico cloning approach guarantees high expression of the MJ-SPIKE vaccine sequence while simultaneously preventing frame shift mutations.

### 3.14. Transformation and Screening of Positive Colonies

The synthetic MJ-SPIKE vaccine construct was transformed into Top10 competent cells. Colonies selected with tetracycline and kanamycin were inoculated in LB media supplemented with both antibiotics. Following plasmid isolation, the primers used were designed to amplify the 381-bp gene fragment. The PCR products were subsequently analyzed on a 1% agarose gel stained with ethidium bromide and visualized using a UV transilluminator, as depicted in Figure S6.

Following the transformation of the Top10 cells, the synthetic MJ-SPIKE vaccine construct was transformed into chemically modified BL21 (D3) cells. After plasmid isolation, the designed primer set was again used for the amplification of the desired fragment. A 381-bp amplicon was observed on a 1% TAE agarose gel and visualized using a UV transilluminator (Figure S7).

### 3.15. Induction of BL21 Cell Culture With IPTG and Western Blot Analysis Using Histidine-Tag Polyclonal Antibodies

**Figure 11:**
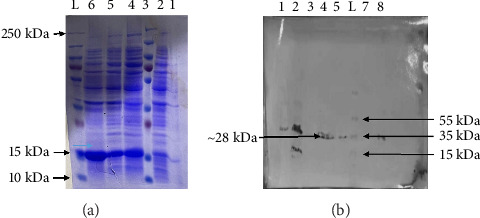
Polyacrylamide gel electrophoresis and Western blotting. (a) 12.5% SDS-PAGE gel showing high expression of the vaccine protein after IPTG induction. (b) Western blot showing the interaction of His tag polyclonal antibodies with our vaccine protein (antigen).

After IPTG induction in the BL21 cell culture, two 12.5% SDS-PAGE reactions were performed. One of the gels was used to determine the expression of the target MJ-SPIKE vaccine protein, and the other was utilized to shift the proteins on the PVDF membrane.  Figure 11(a) shows the gel profile after staining with Coomassie brilliant blue dye, and Figure 11(b) shows proteins that had migrated to the PVDF membrane for Western blotting using polyclonal anti-histidine antibodies. After the color reaction, our MJ-SPIKE vaccine protein exhibited bands at 28 kDa after IPTG induction and cell lysis, as shown in Figure B. The appearance of the approximate 28 kDa band showed that our MJ-SPIKE vaccine protein was expressed as a dimer.

## 4. Discussion

The worldwide impact of the COVID-19 pandemic has raised the need to continue the search for effective vaccines and therapeutic targets [53]. With the development of bioinformatics tools and other computational approaches, peptide-based vaccines are becoming increasingly significant in vaccine development efforts [54]. These peptide-based vaccines have shown efficacy against several viruses including chikungunya, rhinovirus, SLE virus, and dengue virus [55]. However, the mutation rates of some viruses including the SARS-CoV-2 can pose a significant challenge, often leading to immune evasion, and resistance [56]. To address this, our research targeted the S protein, which is a crucial viral component known for its strong antigenicity, nontoxicity, and well-defined three-dimensional structure [57].

Bioinformatics tools are critical in modern vaccine development, especially regarding generating and analyzing protein sequences such as the S protein. This study demonstrated the potential of creating a subunit vaccine that enhances cell-mediated immunity through in silico methods followed by expression in a prokaryotic system [58]. The use of bioinformatics tools and computational approaches could accelerate the vaccine designing and precise selection of the epitopes. These approaches are critical in the run for overcoming the antigenic variability of the virus [59]. While traditional vaccine development has often centered on B-cell responses, recent advancements in computational biology have shifted the focus toward T cells, particularly those involving MHC molecules and HLA systems. T-cell immunity is crucial for generating durable immune responses, which are crucial for successful vaccination [60].

Translating an epitope into a vaccine involves stringent criteria, with the selection of CTL epitopes being a key step. Validating these epitopes for antigenicity using tools such as VaxiJen v2.0 ensures their effectiveness. Additionally, assessing the toxicity of epitopes with ToxinPred is vital for avoiding unintended immune reactions [61]. Besides these, the evaluation of the physiochemical properties of the vaccine is also crucial to predict and ensure feasibility and practicality in the real-world applications and the computational analysis ensures that the vaccine is stable and potent [62]. There have been concerns about certain vaccine types, such as those containing whole viruses or virus-like particles (VLPs), potentially causing adverse effects such as pulmonary immunopathology. Therefore, factors such as antigenicity, toxicity, immunogenicity, and allergenicity are crucial when designing vaccines [63].

This research employed an immunoinformatic methodology to design a vaccine based on CTL epitopes, focusing on structural proteins, particularly the S glycoprotein. The sequence of the vaccine was carefully selected to enhance immunogenicity and stability, and the vaccine exhibited desirable properties, including a stable and hydrophilic composition [64]. Molecular docking studies revealed strong interactions between the vaccine and key receptors, such as TLR-4, TLR-8, and MHC-1. Specifically, the subunit vaccine demonstrated robust binding to the TLR-8 receptor, with a minimal energy value of −1062.4, and favorable interactions with MHC-1, with an energy minimum of −836.9. These findings suggest that the vaccine could induce a potent immune response [65]. These interactions predicted that our vaccine would illicit an effective immune response, and additional comparative analyses could complement and deepen the understanding of its immunogenic potential.

While molecular docking provides valuable insights into protein–protein interactions, it captures only a snapshot of complex physiological dynamics [66]. Therefore, further studies in more flexible environments are necessary to fully understand these interactions. Molecular dynamics simulations further confirmed the stability of the vaccine model, supporting its potential for further development [67]. Following successful in silico amplification, the vaccine sequence was expressed in an *E. coli* host system, specifically using the Top10 and BL21 strains, which are known for their high expression efficiency. Upon induction with IPTG in BL21 cells, the vaccine protein was successfully expressed, as confirmed by SDS-PAGE and Western blot analyses. The consistent results in the expression analysis of our vaccine prove its reliability, further supporting its potential for production at large scale. Future optimizations in fermentation and purification processes could further enhance its yield and commercial feasibility. All these results demonstrate the robust expression and antigenicity of the vaccine, laying the groundwork for additional in vitro and in vivo testing to assess its potential as a COVID-19 therapeutic.

## 5. Conclusion

This study leveraged in silico methods to design a promising COVID-19 vaccine candidate. This approach offers a cost-effective and rapid path to vaccine development with a low failure rate. Our computational work using SnapGene and the pET28b (+) plasmid, in combination with in vitro expression in bacterial systems, confirmed robust protein expression and antigenicity, as evidenced by Western blot analysis showing dimeric forms. While these results are encouraging, particularly given the success of peptide-based vaccines in eliciting strong immune responses, further laboratory validation is essential to confirm the efficacy of these vaccines against COVID-19. This research represents a significant stride toward addressing the ongoing global health crisis.

## Figures and Tables

**Table 1:** Prediction and assessment of CTL epitopes.

No	Epitopes	Antigenicity	Toxicity	Allergenicity	Results
1	VLPFNDGVY	0.4642 (antigen)	Nontoxic	Nonallergen	Selected
2	WTAGAAAYY	0.6306 (antigen)	Nontoxic	Nonallergen	Selected
3	GAAAYYVGY	0.6604 (antigen)	Nontoxic	Nonallergen	Selected
4	ITDAVDCAL	0.5260 (antigen)	Nontoxic	Nonallergen	Selected
5	STQDLFLPF	0.6619 (antigen)	Nontoxic	Nonallergen	Selected

**Table 2:** Conservation prediction of the final selected epitopes of the vaccine construct.

No	Epitope name	Epitope sequence	Length	Percent of protein sequence matches at identity ≤ 100%	Minimum identity (%)	Maximum identity (%)	Selection
1	NP 1	VLPFNDGVY	9	0.00% (0/1)	33.33	33.33	Not selected
2	NP 2	WTAGAAAYY	9	100.00% (1/1)	100.00	100.00	Selected
3	NP 3	GAAAYYVGY	9	100.00% (1/1)	100.00	100.00	Selected
4	NP 4	ITDAVDCAL	9	100.00% (1/1)	100.00	100.00	Selected
5	NP 5	STQDLFLPF	9	0.00% (0/1)	33.33	33.33	Not selected

## Data Availability

Data are available within the article and Supporting Information.

## Supporting Information

Figure S1: World Coverage of Selected Epitopes of MHC Class 1. Figure S2: Protein-Sol Server Predicting the Solubility of the Vaccine. Figure S3: ProSA-WEB showing Z-Score value. Z-score that the ProSA-web server determined was -6.2. Figure S4: Potential Cleaving Sites in the Sequence along with *Nde*I and *Bam*HI. Figure S5: In silico PCR Amplification of target sequence using designed primers. Figure S6: Agarose gel electrophoresis 1% Agarose Gel Showing Amplicon of size 381 bp. Figure S7: Agarose gel electrophoresis 1% Agarose Gel Showing Amplicon of size 381 bp. Table S1: Reaction mixture for PCR amplification of our target sequence. Table S2: PCR Temperature conditions for amplification. Table S3: Recipe mixture for 12.5% resolving gel for SDS-PAGE. Table S4: Recipe mixture for 4% stacking gel for SDS-PAGE. Table S5: The antigenicity prediction of SARS-CoV-2 Surface Glycoprotein. Table S6: Evaluation of the selected proteome. Table S7: Physicochemical Properties, Toxicity, Allergenicity, and Antigenicity Assessment of the Peptide Vaccine. Table S8: Prediction of dissociation constant (Kd) and binding affinity values of the TLR-4, TLR-8, and MHC1 receptors docked complexes by the PRODIGY server.
