# Supplementary material for: Immunotherapeutic Approach for Improving the Efficacy of a Novel Subunit Vaccine Against SARS-CoV-2 by Cytotoxic T-Lymphocytes (CTL) Epitopes
Source: Scientifica (Cairo). 2025 May 26;2025:6025826. doi: 10.1155/sci5/6025826 (PMC12129616; doi:10.1155/sci5/6025826)
Supplement: Supporting Information — Additional supporting information can be found online in the Supporting Information section. [file 6025826.f1.zip › Tables Supplementary.docx]

**Supplementary Tables**

**Tab.S1: Reaction mixture for PCR amplification of our target sequence.**

| **Reaction Mixture** | **Volume** |
| --- | --- |
| Plasmid DNA Template | 2µl |
| PCR Buffer (10X) | 5µl |
| 25mM MgCl2 | 2 µl |
| 10pmol F primer GMJ-80-F | 2 µl |
| 10pmol R primer GMJ-80-R | 2 µl |
| 2mM DNTPs | 2 µl |
| Taq Polymerase (5U/µl) | 1 µl |
| Water | 4 µl |
| **Total Volume** | **20 µl** |

**Tab.S2: PCR Temperature conditions for amplification**

| **Temperature** | **Time** |
| --- | --- |
| Initial denaturation temperature: 95°C | 5 mins |
| Denaturation Temperature: 95°C | 30 secs |
| Annealing Temperature: 52°C | 30secs |
| Extension Temperature: 72°C | 1 min |
| Final Extension Temperature: 72°C | 10mins |
| Hold Temperature: 4°C | ∞ |

**Tab.S3: Recipe mixture for 12.5% resolving gel for SDS-PAGE**

| **Resolving Gel** | **Volume** |
| --- | --- |
| H_2_0 | 4.71ml |
| 1.5M Tris-Cl buffer (pH 8.8) | 3.75ml |
| 30% acrylamide | 6.24ml |
| 10% SDS | 150ul |
| 10% APS | 150ul |
| TEMED | 15ul |

**Tab.S4: Recipe mixture for 4% stacking gel for SDS-PAGE**

| **Stacking Gel** | **Volume** |
| --- | --- |
| H_2_0 | 4.08ml |
| 1 M Tris-Cl buffer (pH 6.8) | 750ul |
| 30% acrylamide | 1020ul |
| 10% SDS | 60ul |
| 10% APS | 60ul |
| TEMED | 5ul |

**Tab.S5: The antigenicity prediction of SARS-CoV-2 Surface Glycoprotein**

| **Protein** | **Accession number** | **Amino Acids** |
| --- | --- | --- |
| SARS-CoV-2 Surface Glycoprotein | QZH77230.1 | 1273 aa |

**Tab.S6: Evaluation of the selected proteome.**

| **Antigenicity** | 0.4688 (Probable ANTIGEN) |
| --- | --- |
| **Allergenicity** | Non-Allergen |
| **Toxicity** | Non-Toxic |
| **Number Of Amino Acids** | 1273 |
| **Molecular Weight** | 141264.23 |
| **Theoretical pI** | 6.64 |
| **Estimated Half Life** | >10 Hours (Escherichia Coli, In Vivo) |
| **Instability Index** | 32.89 (The Protein Is Stable) |
| **Aliphatic Index** | 84.37 |
| **Grand Average Of Hydropathicity (GRAVY)** | -0.089 |

**Tab.S7: Physicochemical Properties, Toxicity, Allergenicity, and Antigenicity Assessment of the Peptide Vaccine.**

| **Property** | **Value** |
| --- | --- |
| **Number of amino acids** | 122 |
| **Molecular weight** | 13648.51kDa |
| **Chemical formula** | C_623_H_955_N_159_O_182_S_2_ |
| **Theoretical pI** | 5.56 |
| **Total number of negatively charged residues (Asp + Glu)** | 13 |
| **Total number of positively charged residues (Arg + Lys)** | 11 |
| **Total number of atoms** | 1921 |
| **Instability index** | 26.11 |
| **Aliphatic index** | 91.97 |
| **GRAVY** | -0.172 |
| **Antigenicity** | VaxiJen: (antigenic) |
| **Allergenicity** | Non-allergen |
| **Toxicity** | Non-toxic |

**Tab.S8: Prediction of dissociation constant (Kd) and binding affinities values of the TLR4, TLR8 and MHC1 receptors docked complexes by PRODIGY server.**

| **Properties** | **TLR-4 Receptor** | **TLR-8 Receptor** | **MHC-1 Receptor** |
| --- | --- | --- | --- |
| **ΔG (kcal mol-1)** | -8.0 | -14.0 | -11.9 |
| **Kd (M) at ℃** | 2.3e-06 | 1.3e-10 | 4e-09 |
| **ICs charged-charged.** | 9 | 25 | 12 |
| **ICs charged-polar.** | 9 | 26 | 10 |
| **ICs charged apolar** | 5 | 21 | 21 |
| **ICs polar-polar** | 6 | 3 | 5 |
| **ICs polar apolar** | 6 | 18 | 20 |
| **ICs apolar apolar** | 5 | 53 | 21 |
| **NIS charged** | 25.67 | 27.74 | 34.72 |
| **NIS apolar** | 31.73 | 32.47 | 32.29 |
