# Supplementary figures and images for: Immunotherapeutic Approach for Improving the Efficacy of a Novel Subunit Vaccine Against SARS-CoV-2 by Cytotoxic T-Lymphocytes (CTL) Epitopes
Source: Scientifica (Cairo). 2025 May 26;2025:6025826. doi: 10.1155/sci5/6025826 (PMC12129616; doi:10.1155/sci5/6025826)

# Population: World

| MHC class | Coverage | Average hit | PC90 |
|-----------|----------|-------------|------|
| I         | 25.61%   | 0.27        | 0.13 |

World - Class I Coverage

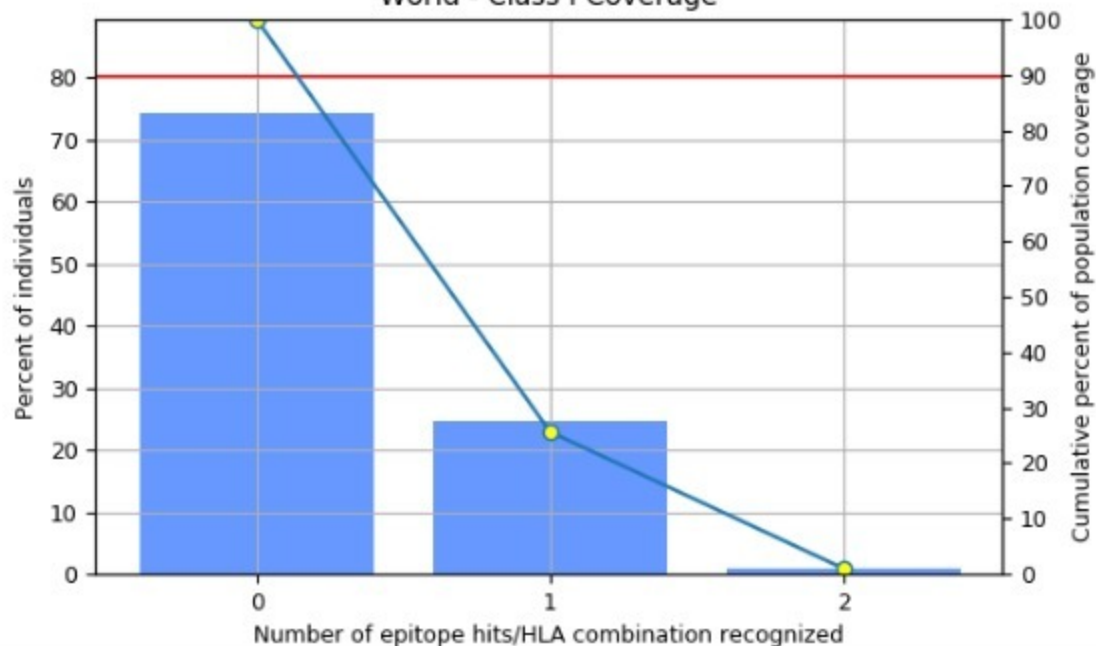

Supplement: Supporting Information — Additional supporting information can be found online in the Supporting Information section. [file 6025826.f1.zip › Fig.S1.pdf]

# Solubility

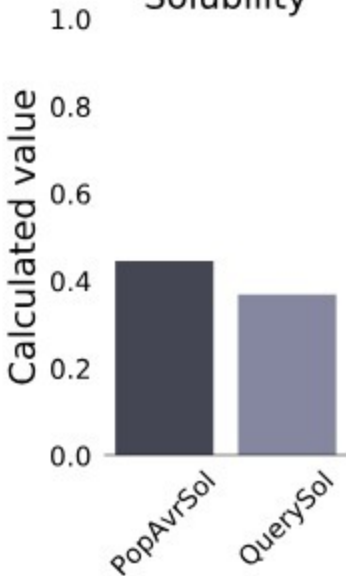

Supplement: Supporting Information — Additional supporting information can be found online in the Supporting Information section. [file 6025826.f1.zip › Fig.S2.pdf]

# Overall model quality

Z-Score: **-6.2**

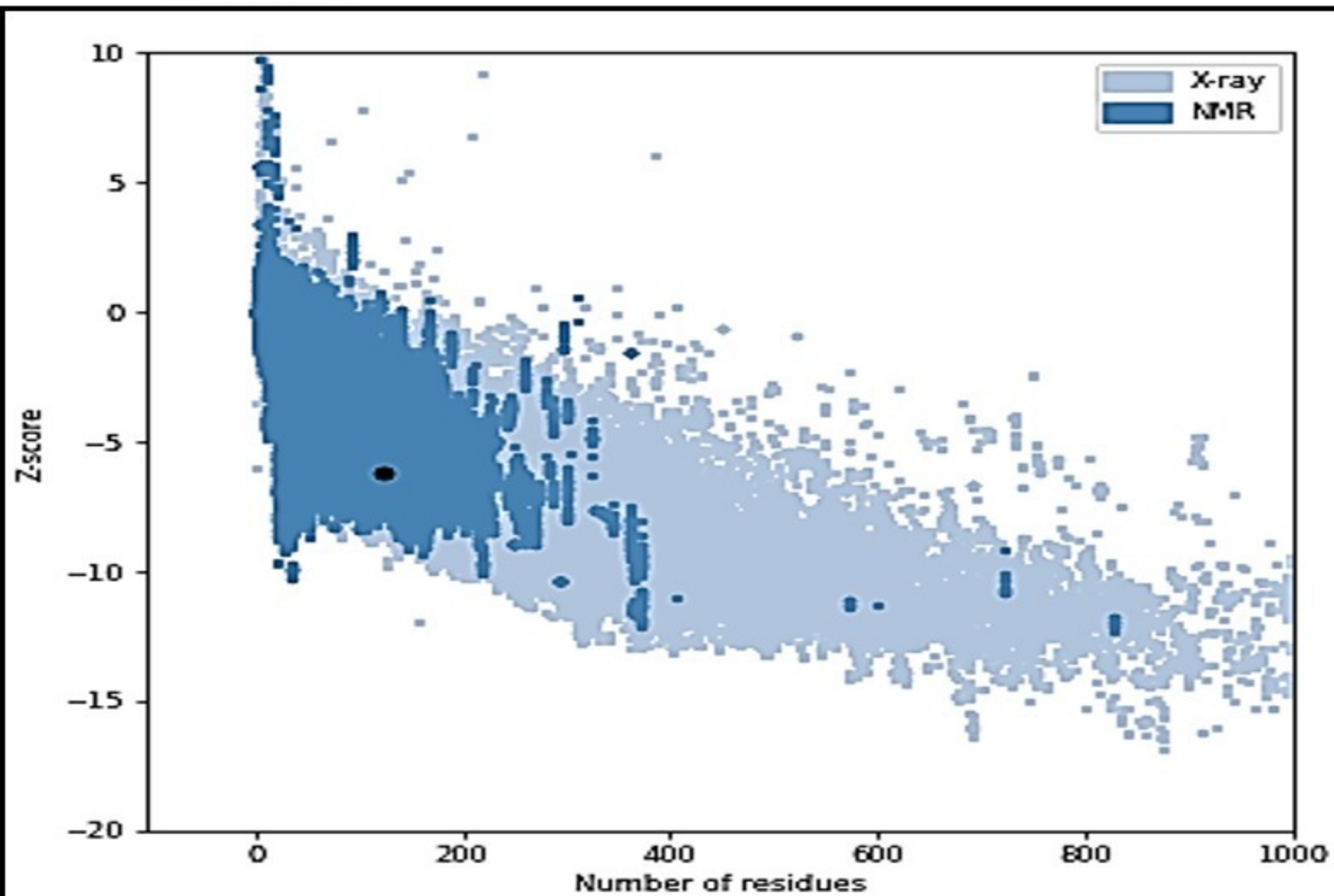

Supplement: Supporting Information — Additional supporting information can be found online in the Supporting Information section. [file 6025826.f1.zip › Fig.S3.pdf]

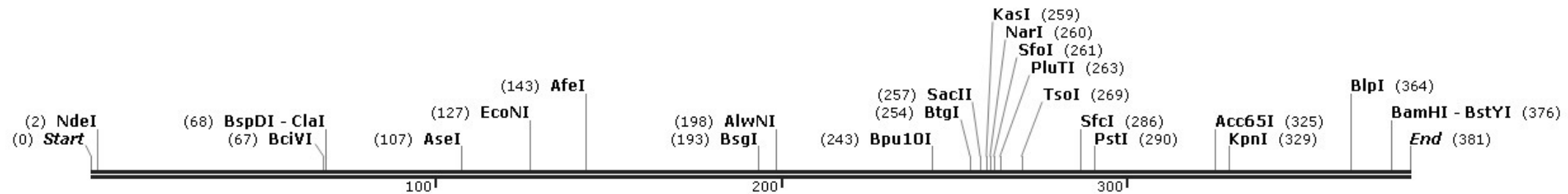

selected vaccine sequence with restriction sites Nde1 and BamH1

381 bp

Supplement: Supporting Information — Additional supporting information can be found online in the Supporting Information section. [file 6025826.f1.zip › Fig.S4.pdf]

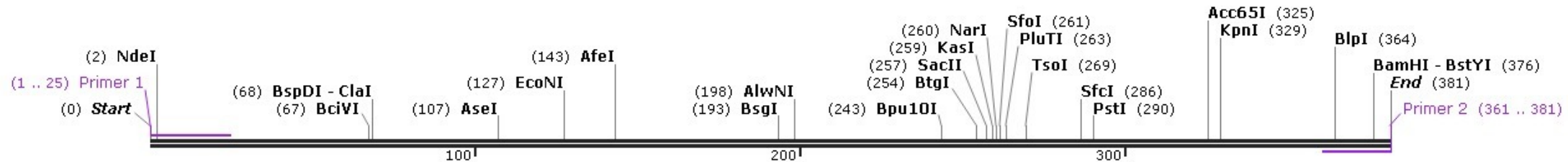

Amplified  
381 bp

Supplement: Supporting Information — Additional supporting information can be found online in the Supporting Information section. [file 6025826.f1.zip › Fig.S5.pdf]

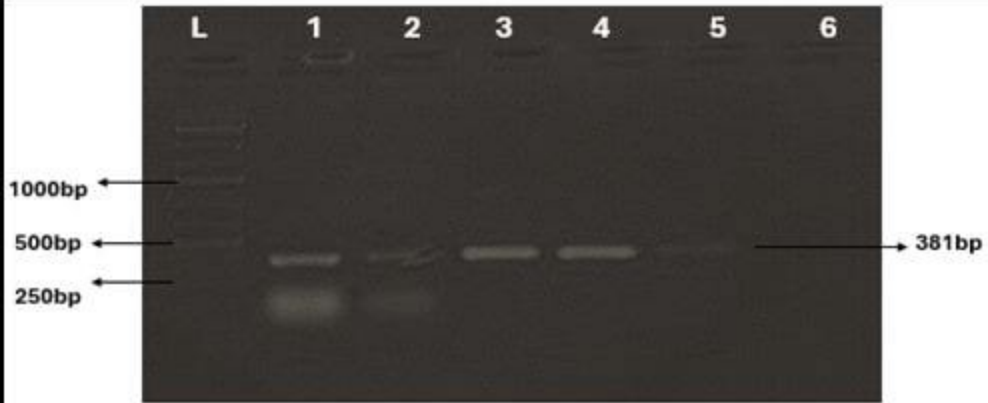

(a)

Supplement: Supporting Information — Additional supporting information can be found online in the Supporting Information section. [file 6025826.f1.zip › Fig.S6.pdf]

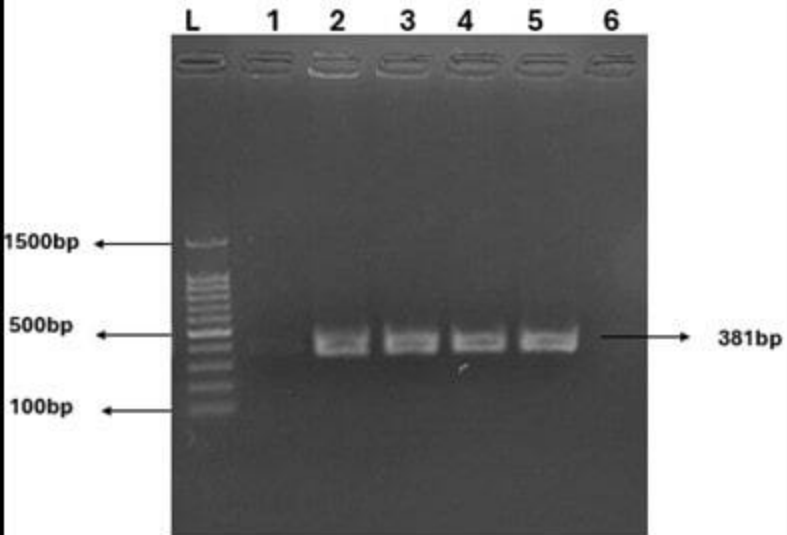

(a)

Supplement: Supporting Information — Additional supporting information can be found online in the Supporting Information section. [file 6025826.f1.zip › Fig.S7.pdf]
